# FCCD-SAR: A Lightweight SAR ATR Algorithm Based on FasterNet

**DOI:** 10.3390/s23156956

**Published:** 2023-08-05

**Authors:** Xiang Dong, Dong Li, Jiandong Fang

**Affiliations:** The College of Information Engineering, Information and Communication Engineering, Inner Mongol University of Technology, Hohhot 010080, China

**Keywords:** synthetic aperture radar (SAR), automatic target recognition (ATR), deep learning, lightweight, fasterNet

## Abstract

In recent times, the realm of remote sensing has witnessed a remarkable surge in the area of deep learning, specifically in the domain of target recognition within synthetic aperture radar (SAR) images. However, prevailing deep learning models have often placed undue emphasis on network depth and width while disregarding the imperative requirement for a harmonious equilibrium between accuracy and speed. To address this concern, this paper presents FCCD-SAR, a SAR target recognition algorithm based on the lightweight FasterNet network. Initially, a lightweight and SAR-specific feature extraction backbone is meticulously crafted to better align with SAR image data. Subsequently, an agile upsampling operator named CARAFE is introduced, augmenting the extraction of scattering information and fortifying target recognition precision. Moreover, the inclusion of a rapid, lightweight module, denoted as C3-Faster, serves to heighten both recognition accuracy and computational efficiency. Finally, in cognizance of the diverse scales and vast variations exhibited by SAR targets, a detection head employing DyHead’s attention mechanism is implemented to adeptly capture feature information across multiple scales, elevating recognition performance on SAR targets. Exhaustive experimentation on the MSTAR dataset unequivocally demonstrates the exceptional prowess of our FCCD-SAR algorithm, boasting a mere 2.72 M parameters and 6.11 G FLOPs, culminating in an awe-inspiring 99.5% mean Average Precision (mAP) and epitomizing its unparalleled proficiency.

## 1. Introduction

Synthetic aperture radar (SAR) [1] has achieved extensive utilization in diverse fields such as reconnaissance detection, geological exploration, disaster detection, and public area security screening. Its ability to operate round the clock and in all weather conditions makes it a vital tool. Automatic target recognition (ATR) [2] plays a crucial role in SAR image interpretation, encompassing the recognition of target regions of interest and inference of target class attributes. This paper focuses on SAR-ATR, which holds immense practical value and theoretical significance, providing valuable insights for image recognition [3], target recognition [4], image matching [5], and other remote sensing applications.

SAR achieves high-resolution imaging through linear FM signals and matched filtering techniques for distance direction, while the azimuth direction employs motion-based virtual aperture synthesis. It possesses remarkable attributes such as all-day [6], all-weather operability [7], high penetration capability [8], long-range observation [9], and high-resolution ground imaging [10]. The wealth of surface electromagnetic scattering information offered by SAR has contributed to its widespread adoption in various domains, including ocean exploration [11], forestry census [12], topographic mapping [13], land resource survey, and traffic control, as well as military applications like battlefield reconnaissance, radar guidance, and strike effect evaluation [14]. Enhancing image quality plays a crucial role in SAR applications, encompassing image super-resolution, denoising, deblurring, and contrast enhancement. These techniques not only improve visual effects but also enhance feature extraction quality, thereby facilitating subsequent image understanding and interpretation. However, traditional SAR imaging algorithms face challenges in effectively detecting and imaging moving targets due to their complex scattering characteristics and motion features.

With the rapid advancements in deep learning, convolutional neural networks (CNNs) have been increasingly employed for ship recognition in SAR images, exhibiting promising outcomes. However, deeper CNNs focus on accuracy at the expense of real-time performance, speed, and compatibility with resource-constrained embedded platforms. Hence, there is a pressing need to develop lightweight models that strike a balance between speed and accuracy, enabling real-time ship target recognition in SAR images and seamless deployment on embedded platforms [15]. In the realm of traditional machine learning, feature extraction and classification algorithms are commonly used for target recognition. Feature extraction techniques include edge, texture, shape, and polarization characteristics, enabling the extraction of target feature information from SAR images. The wavelet transform, Gabor filter, grayscale co-occurrence matrix, and principal component analysis are popular feature extraction algorithms. Following feature extraction, classifiers such as support vector machines (SVMs) [16], artificial neural networks, and decision trees are utilized for target classification. Traditional machine learning methods also encompass feature matching-based approaches, such as polarized scattering similarity and adaptive local orientation patterns. Du proposed the Fast C&W algorithm to counter attacks on SAR target recognition by deep convolutional neural networks [17]. Peng proposed a Speckle-variant attack algorithm for the adversarial attack on SAR target recognition of deep convolutional neural networks [18].

While traditional machine learning methods can achieve satisfactory results in SAR target recognition, they have limitations. Subjectivity and incompleteness arise from the need for manual feature selection during extraction. Furthermore, the performance of traditional machine learning methods is constrained by the capabilities of feature extraction and classifiers.

In recent years, the emergence of deep learning has prompted researchers to explore its application in SAR target recognition. Two-stage target recognition methods generally offer higher accuracy compared to single-stage methods. However, two-stage algorithms often exhibit slower training and recognition speeds compared to their single-stage counterparts. To address this limitation, researchers have increasingly turned to single-stage algorithms to ensure real-time recognition. However, single-stage methods are more susceptible to false recognitions and localization errors, particularly for small targets. Thus, there is a need to enhance the performance of single-stage algorithms in detecting small targets in real-time applications.

Existing target recognition algorithms primarily cater to optical images, focusing on accuracy improvement. Few detectors have been specifically tailored for SAR images. Directly applying target recognition algorithms designed for optical images to SAR images may yield suboptimal results due to differences in imaging mechanisms, target characteristics, and resolution disparities. Therefore, it is crucial to develop target recognition algorithms that consider the unique complexities and characteristics of SAR images.

Given the extensive SAR image data and high feature dimensionality, traditional deep learning models often suffer from a large number of parameters and high computational complexity. Hence, the research focus has shifted toward lightweight networks. Lightweight networks refer to neural network structures that achieve computational efficiency by reducing parameters and computations. In SAR automatic target recognition, lightweight networks offer improved computational speed and memory efficiency without compromising classification accuracy.

In conclusion, the demand for a lightweight and readily deployable single-stage target recognition algorithm for SAR image target recognition, especially on embedded platforms, is becoming increasingly urgent. This paper introduces the FCCD-SAR method, which addresses the SAR image target recognition challenge by striking a well-balanced approach. Our approach significantly improves recognition accuracy while minimizing the number of parameters and floating-point operations (FLOPs) required. The main contributions of this paper can be summarized as follows.

(1) To facilitate the development of a SAR image target recognition algorithm that is well-suited for embedded platforms, we adopt a more rational approach by employing the FasterNet algorithm for dataset lightening and feature extraction. This enables better alignment with the unique characteristics of SAR image data. Moreover, we effectively reduce the number of parameters while preserving satisfactory performance.

(2) To enhance the extraction of scattering information from targets and improve target recognition accuracy, we propose the utilization of a lightweight upsampling operator called CARAFE. This operator exhibits a wide perception field during reconfiguration, allowing for effective improvement in the recognition performance of SAR targets. Additionally, this design enables a reduction in the number of parameters and floating-point operations (FLOPs) required while maintaining high recognition performance.

(3) To further improve the model’s recognition accuracy and computational efficiency, a faster and lighter module C3-Faster is used to reduce the number of parameters and computation while ensuring recognition accuracy.

(4) For the characteristics of multi-scale and large-scale variation of SAR targets, DyHead’s attention-based mechanism detection head is used to better detect feature information at different scales adequately and improve the recognition effect of SAR targets.

(5) To obtain the ultimate network model, a pruning operation is introduced to prune the network structure to obtain the minimum optimal network model with guaranteed accuracy.

## 2. Related Work

Numerous publicly available datasets exist for SAR target recognition, with the MSTAR ten-class classification dataset being the most renowned. Various algorithms, including traditional and deep learning-based approaches, have been employed for SAR target recognition. Traditional algorithms often prove ineffective, relying heavily on manual parameter setting and design, lacking robustness, and exhibiting poor generalization to other SAR datasets. Additionally, their recognition speed and real-time performance fall short of engineering application requirements.

Consequently, the benefits of end-to-end deep learning algorithms have become increasingly evident. In recent years, researchers have shifted towards deep learning algorithms for SAR image target recognition, capitalizing on advancements in deep learning techniques. These algorithms eliminate the need for intricate manual feature extraction, instead focusing on designing robust network structures to effectively extract SAR target features. Convolutional neural networks (CNNs) have gained significant popularity in SAR image target recognition, particularly for ship targets. Pre-trained CNN [19] models have yielded promising results for feature extraction in SAR images, followed by classification using traditional classifiers. With the continuous evolution of deep learning techniques, researchers have explored complex deep neural network models such as RNNs and graph convolution neural networks (GCNNs) [20] for SAR target-recognition tasks.

The development of deep learning algorithms has introduced new methods for SAR target recognition. For instance, region extraction algorithms commonly used in target recognition, such as region-based convolutional neural networks (R-CNN) [21] and Single Shot MultiBox Detectors (SSD) [22], have been adapted for SAR target recognition. Additionally, novel network architectures and techniques have been proposed, such as the feature pyramid network for target recognition [23] and the fully convolutional attention block algorithm for SAR target recognition [24]. However, these methods often rely on deep network structures without considering practical engineering applications, leading to imbalanced parameters, FLOPs, and recognition accuracy.

Improving the accuracy and robustness of target recognition can be achieved by fusing SAR images with data from multiple sources, such as optical and infrared images. Multi-source data fusion plays a crucial role in SAR automatic target recognition [25], utilizing information from various sources for comprehensive analysis [26] and feature extraction to enhance accuracy and robustness. Data-level fusion and feature-level fusion are two main aspects of multi-source data fusion [27].

Given the growing interest in lightweight SAR target-recognition models, the focus has shifted toward networks with reduced parameters and computational requirements. Lightweight networks offer improved computational speed and memory efficiency without sacrificing classification accuracy [28]. Notable lightweight designs include a lossless lightweight CNN proposed by Zhang [29] and a modified convolutional random vector function link network [30] for SAR target recognition. However, existing models often fail to strike the appropriate balance between accuracy and lightweight design and neglect the need to tailor recognition models specifically for SAR image target recognition datasets.

Therefore, to address the requirements of real-world engineering applications, we have devised an innovative SAR target recognition algorithm that is both lightweight and highly precise. This algorithm has been specifically designed to cater to SAR image target recognition datasets.

## 3. Materials and Methods

In this paper, we propose a lightweight SAR ART algorithm based on FasterNet, the FCCD-SAR. As a consequence, we strike the best balance between accuracy and lightweight design. The FCCD-SAR model mainly consists of the following modules and strategies: the state-of-the-art target-recognition benchmark framework YOLOV5, the faster neural network backbone network FasterNet, the lightweight upsampling operator CARAFE that solves the problems of some general modules and operators, the faster lightweight module C3- Faster, and DyHead, which uses an attention mechanism to unify different target-detection heads. Compared to the current state-of-the-art methods, such as YOLOV8 and YOLOV7, YOLOV8 has just been released recently, and its model is still in the stage of frequent modification, thus it is not stable enough. YOLOV7, on the other hand, has a slightly lower inference speed than YOLOV5 and requires more memory resources. In contrast, YOLOV5, after many official modifications, has a more stable performance, a more mature network, and a faster inference speed, and at the same time, it is more economical in terms of memory consumption. Therefore, YOLOV5 was chosen as the benchmark framework for this study.

### 3.1. Architectural Overview of FCCD-SAR Network

**Figure 1:**
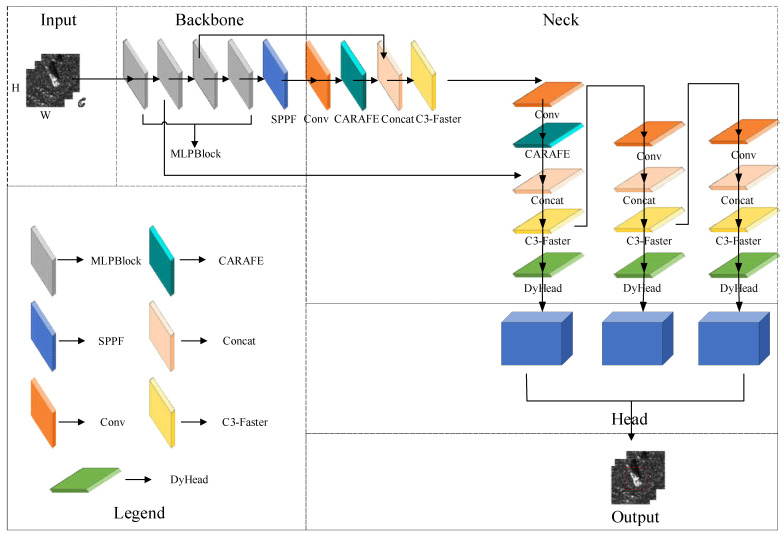
The overall network architecture of the FCCD-SAR model. There are five parts in the network structure, and the contents of each part are shown in the figure. SPPF: Spatial Pyramid Pooling-Fast.

**Figure 2:**
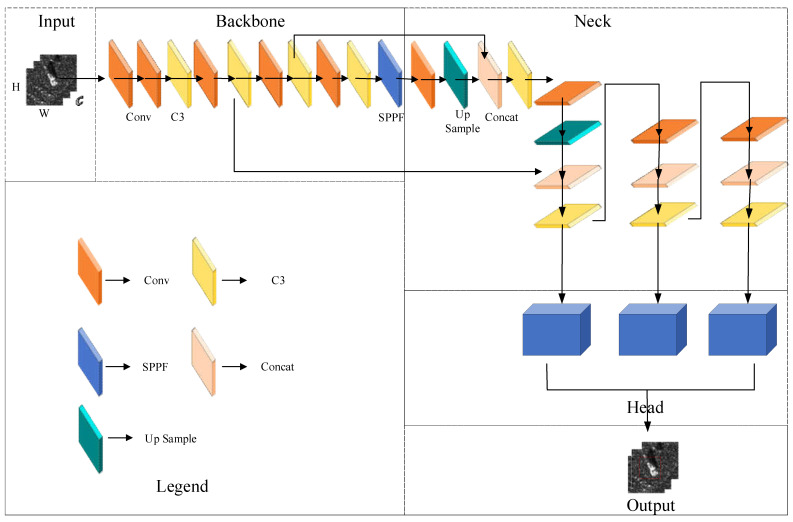
Basic YOLOV5 network framework.

The schematic representation in Figure 1 depicts the holistic network architecture of our FCCD-SAR model. Figure 2 shows the basic YOLOV5 network framework diagram, which facilitates the comparison with the improved structure. The model depicted in the figure comprises five distinct components: input, backbone, neck, head, and output. Notably, the input image initially undergoes processing through FasterNet [31], a custom-designed lightweight backbone network. This backbone network is adept at extracting the discrete scattering features of SAR images more rationally.

Then, the backbone network into the neck meets the lightweight, universal upsampling operator CARAFE [32], which is used to achieve significant improvements in different tasks while introducing only a small number of parameters and computational costs. Then, before extracting the small target scale features output, a faster lightweight module C3-Faster is introduced to combine the respective advantages of CNN and self-attention to complement each other, which can improve the recognition performance of SAR targets while reducing the number of parameters and FLOPs. Finally, the processed image flows into DyHead [33], our chosen attention-based detection head. DyHead incorporates attention mechanisms across scale-aware feature layers, spatial locations for spatial perception, and output channels for task perception. This innovative approach greatly enhances the expressiveness of the model’s target-detection head without imposing an additional computational burden. We will discuss the detailed improvements in these four areas later on.

### 3.2. Faster and Better Neural Networks: FasterNet

To design fast neural networks, much work has focused on reducing the number of FLOPs. However, we observe that this reduction in FLOPs does not necessarily lead to a similar degree of reduction in latency. This mainly stems from the inefficiency of low FLOPS per second.

To achieve faster networks, we revisited the popular operators and demonstrated that such low FLOPS are mainly due to frequent memory accesses of the operators, especially deep convolution. Therefore, we adopted a new partially convolutional (PConv) that can extract spatial features more efficiently by reducing both redundant computations and memory accesses.

To optimize the backbone network, we incorporated FasterNet-T0, the smallest version of FasterNet, and retained its MLPBlock architecture. Additionally, we made improvements by eliminating unnecessary MLPBlocks through stacking. The resulting lightweight backbone, FasterBackbone, was specifically designed to efficiently extract scattering features from SAR datasets.

**Figure 3:**
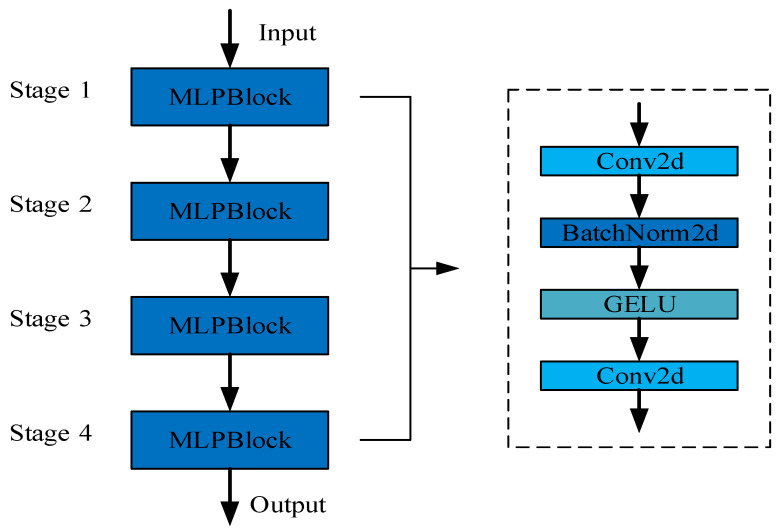
Details of the FasterBackbone network structure. The FasterBackbone consists of a total of four MLPBlocks, and the specific structure of the MLPBlock is shown in Figure 3. GELU: Gaussian Error Linear Unit.

Figure 3 provides an overview of the structural details of FasterBackbone, while Table 1 presents the specific parameters used. Through extensive experimental validation using the SAR dataset, we demonstrated the remarkable feature extractability of the backbone we designed.

### 3.3. Lightweight Upsampling Operator: CARAFE

The upsampling operation was achieved through feature recombination, which involves the dot product between the upsampling kernel and the corresponding neighborhood pixels in the input feature map. The fundamental network structure, with a small receptive field, ignores some useful information, and therefore, the receptive field needs to be enlarged. The upsampling operation CARAFE, on the other hand, can have a large receptive field during reorganization and guides the reorganization process based on the input features. Meanwhile, the whole CARAFE operator structure is small, which meets the lightweight requirement. Specifically, the input feature map is utilized to predict unique upsampling kernels for each position, followed by feature recombination based on these predicted kernels. CARAFE demonstrates significant performance improvements across various tasks while only introducing minimal additional parameters and computational overhead.

**Figure 4:**
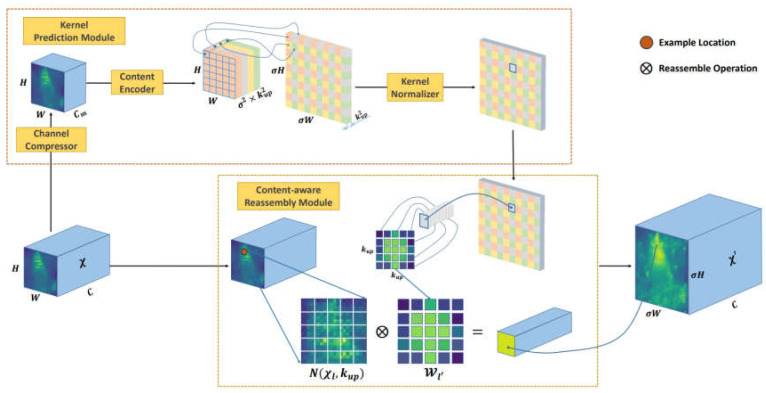
The overall framework of CARAFE. CARAFE is composed of two key components, i.e., kernel pre-diction module and content-aware reassembly module. A feature map with size C × H × W is upsampled by a factor of σ (=2) in this figure.

CARAFE consists of two primary modules: the upsampling kernel prediction module and the feature recombination module, as depicted in Figure 4. Assuming an upsampling multiplier of σ and an input features map with dimensions H×W×C, the process begins by predicting the upsampling kernel through the upsampling kernel prediction module. Subsequently, the feature recombination module is employed to complete the upsampling procedure, resulting in an output feature map with dimensions σH×σW×C.

Given an input feature map of shape H×W×C, our initial step involves channel compression, reducing the channel number to $C_{m}$ using a 1×1 operation. The primary objective of this compression is to alleviate the computational burden on subsequent steps. Following that, we proceeded with content encoding and upsampling kernel prediction, assuming a specific upsampling kernel size of $k_{up}\times k_{up}$. It is worth noting that a larger upsampling kernel offers a broader perceptual field, but it also entails a higher computational cost. To incorporate distinct upsampling kernels for each position in the output feature map, it is necessary to predict the shape of the upsampling kernel as $\sigma H\times \sigma W\times k_{up}\times k_{up}$. In the initial step, after compressing the input feature map, we employed a convolutional layer with $k_{encoder}\times k_{encoder}$ channels to predict the upsampling kernel. The number of input channels is $C_{m}$, and the number of output channels is $\sigma^{2}k_{up}^{2}$. Following this, we expanded the channel dimension across the spatial dimension, resulting in an upsampling kernel with the shape $\sigma H\times \sigma W\times k_{up}^{2}$.

At each location within the output feature map, we performed a mapping back to the corresponding region in the input feature map. This region, centered on the location, encompassed a region of size $k_{up}\times k_{up}$. Subsequently, we computed the dot product between this region and the predicted upsampling kernel specific to that point, resulting in the output value. It is worth noting that different channels at the same location shared the same upsampling kernel.

### 3.4. Faster and Lighter Modules: C3-Faster

In recent years, the fields of computer vision (CV) have witnessed a surge of interest in convolutional neural networks (CNNs) and self-attention networks (SNNs). CNNs have achieved remarkable breakthroughs in CV domains, including image classification, target recognition, and target tracking, consistently attaining state-of-the-art performance across diverse datasets. Concurrently, the rapid development of vision transformers has led to the emergence of transformer-based models with various self-attention mechanisms that have begun to surpass CNNs in several vision tasks, thereby redefining the performance benchmarks in these areas.

**Figure 5:**
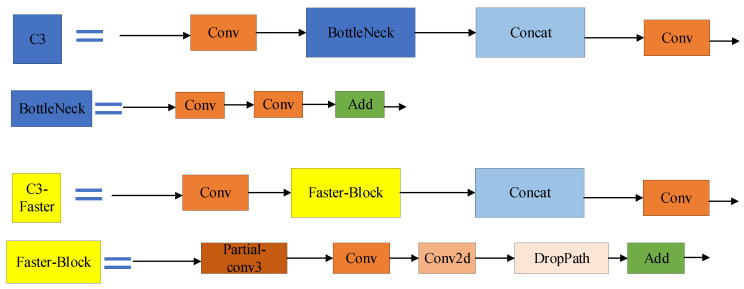
Comparison of C3-Faster of FCCD-SAR and the C3 structure of YOLOV5. Faster-Block and BottleNeck structure are shown in Figure 5.

ACmix offers a compelling fusion of convolution and self-attention, making it a suitable approach for enhancing hybrid representation learning in SAR image target recognition. With the challenge of detecting small targets in SAR images in mind, we opted to replace the original YOLOV5 C3 module with C3-Faster, provided by FasterNet. Faster-Block and BottleNeck structures are shown in Figure 5. Among them, Faster-Block has one more partial convolution than BottleNeck for spatial fusion and one Drop path to reduce the amount of calculation. Figure 5 showcases the design of this faster, lightweight module. By incorporating the lightweight C3-Faster, we further enhanced the speed of target recognition, addressing the need for efficient and swift target identification.

### 3.5. Detection Head Based on Attention Mechanism: DyHead

In Figure 1, the third component showcased our attention mechanism-based detection head called DyHead, which was tailored for the SAR image dataset. DyHead introduced a novel dynamic head framework that unifies various target detection heads using an attention mechanism. By leveraging attention between feature levels for scale perception, spatial locations for spatial perception, and output channels for task perception, this approach substantially enhances the expressiveness of the model’s target detection head without imposing additional computational burden.

DyHead is a fusion of three attention mechanisms: scale-aware attention $\pi_{L}$, spatial attention $\pi_{S}$, and channel attention $\pi_{C}$. These attention mechanisms are stacked together to form a single block. The final head consists of multiple blocks, each incorporating this stack of attention mechanisms.

With the feature tensor $F\in R^{L\times H\times W\times C}$ at hand, we can describe the generalized form of self-attentiveness as follows:

(1) W(F)=π(F)×F

The simplest approach would be to employ a fully connected layer, but directly learning the attention function across all dimensions would result in excessive computational requirements and prove impractical due to the high dimensionality. Instead, we tackled this challenge by breaking down the attention function into three sequential attentions, each targeting a single dimension:

(2) $$W\left(F\right)=\pi_{C}\left(\pi_{S}\left(\pi_{L}\left(F\right)\times F\right)\times F\right)\times F$$

Scale-aware attention $\pi_{L}$: to address the fusion of features at different scales based on their semantic significance, we began by introducing scale-aware attention:

(3) $$\pi_{L}\left(F\right)\times F=\sigma \left(f\left(\frac{1}{SC}\underset{S,C}{\sum }F\right)\right)\times F$$

In this context, f(⋅) corresponds to a linear function that utilizes 1×1 convolutional approximation, while σ(x) represents a hard-sigmoid activation function.

Spatial-aware Attention $\pi_{S}$: continuing with our exploration, we then introduced another module called spatial location-aware attention to emphasize the discriminative capabilities of various spatial locations. Given the large extent of S, we decoupled it into two stages: first, we employed deformation convolution to achieve sparse attention learning, and then we integrated features across different scales to complete the process:

(4) $$\pi_{S}\left(F\right)\times F=\frac{1}{L}\overset{L}{\underset{l=1}{\sum }}\overset{K}{\underset{k=1}{\sum }}w_{l,k}\times F\left(l;p_{k}+\Delta_{p_{k}};c\right)\times \Delta_{m_{k}}$$

In this scenario, *K* represents the number of sparsely sampled positions. The remaining parameter information is analogous to that in deformation convolution, $\omega_{l,k}$ is an importance factor to add bias, and $\Delta m_{k}$ is an importance factor for adaptive weighting and thus, it is omitted for brevity.

Task-aware attention $\pi_{C}$: to facilitate collaborative learning with the enhanced generalizability of goal representation capabilities, we devised a task-aware attention mechanism. This attention mechanism dynamically adjusts feature channels to assist various tasks as needed:

(5) $$\pi_{C}\left(F\right)\times F=\max\left(\alpha^{1}\left(F\right)\times F_{c}+\beta^{1}\left(F\right),\alpha^{2}\left(F\right)\times F_{c}+\beta^{2}\left(F\right)\right)$$

**Figure 6:**
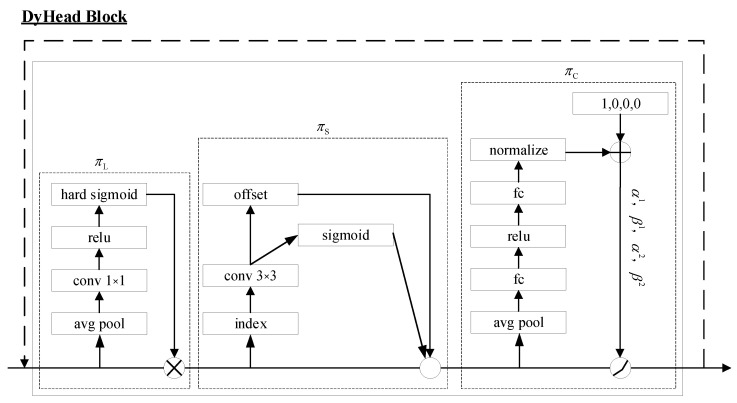
A detailed design of Dynamic Head.

The hyperparameter plays a crucial role in controlling the activation threshold, akin to DyReLU. α and β are used as rescale and reshift, respectively. By sequentially implementing the aforementioned attention mechanism, we can stack multiple instances of it. The configuration of the DynamicHead is illustrated in Figure 6, providing a visual representation of its structure.

### 3.6. Pruning

The deployment of CNNs in practical applications is often hindered by their high computational requirements. In this study, we propose a straightforward and efficient approach called network pruning, which involves the sparsity of network channels. This method is particularly well-suited for CNN architectures, as it minimizes the training overhead and yields models that can be deployed without the need for specialized hardware or software acceleration while still maintaining high performance. By training on thick networks and automatically filtering and removing redundant channels during the training process, we can generate streamlined networks that achieve comparable accuracy levels.

**Figure 7:**
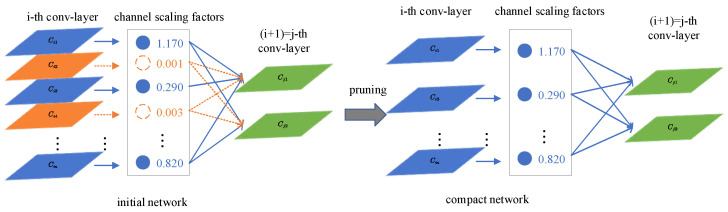
Pruning process diagram.

This process involves applying L1 regularization to the scaling factor within the Batch Normalization (BN) layer and iteratively adjusting the scaling factor. By converging the scaling factor towards zero, we can identify and remove unimportant channels. This can be visualized in Figure 7, where the regularization gradually reduces the scaling factor values, leading to the elimination of unnecessary channels.

By applying L1 regularization to the scaling factors, each corresponding to a specific convolutional channel or neuron in the fully connected layer, we can effectively discriminate and prune unimportant channels in subsequent operations. Although the additional regularization term has a minimal impact on model performance, it can potentially enhance training accuracy. While pruning unimportant channels may initially lead to a temporary performance drop, this can be rectified through subsequent fine-tuning.

The pruned network obtained after the pruning process exhibits a more compact size, reduced running time, and decreased computational operations compared to the original network.

For each channel in the network, a scaling factor γ is introduced, which is multiplied by the output of that channel. Both the network and these scale factors undergo training, and sparse regularization is continuously applied to the scaling factors throughout the training process. Eventually, channels with significantly small scaling factor values are pruned, and the network is further fine-tuned. Here are the specific details of the process:

(6) $$L=\underset{\left(x,y\right)}{\sum }l\left(f\left(x,W\right),\right)+\lambda \underset{\gamma \in \Gamma }{\sum }g\left(\gamma \right)$$

In the training process, the loss function is defined as a combination of terms. The first term on the left represents the loss for the normal training of the CNN, where (*x*, *y*) denotes the input and target. The second term g(⋅) introduces a sparsity penalty on the scaling factor. The balance factor λ normalizes the latter term. In our experiments, the L1 paradigm, g(s)=|s| is chosen for the later sparsification training. To optimize the non-smoothed L1 penalty term, we use the subgradient descent method. Alternatively, the non-smoothed L1 penalty can be replaced with a smoothed L1 penalty to avoid the need for subgradients at non-smoothed points.

Channel pruning involves removing the input-output connectivity related to the channel, leading to a narrower network. The scaling factors serve as channel selectors, and when optimized with the network, they facilitate the removal of unimportant channels without significantly affecting the generalization performance.

BN finds extensive application in contemporary CNNs, facilitating rapid model convergence and enhancing generalization performance. We draw inspiration from BN’s technique of normalizing activation values and employ it as a basis to devise a straightforward yet efficient approach for combining channel scaling factors. Specifically, the BN layer employs mini-batch statistics to normalize the activation values within a given segment. Considering $z_{in}$ as the input and $z_{out}$ as the output of the BN layer, with B representing the present mini-batch. The BN layer executes the subsequent transformation:

(7) $$z=\frac{z_{in}-\mu_{B}}{\sqrt{\sigma_{B}^{2}+\varepsilon }};z_{out}=\gamma z+\beta$$

where $\mu_{B}$ represents the mean and $\sigma_{B}$ represents the variance of the inputs in B. γ and β denote trainable affine transformation parameters responsible for normalizing the activation values and subsequently linearly transforming them to an arbitrary scale.

The conventional approach is to add a BN layer after the convolutional layer with a channel scaling/offset factor. This allows us to directly use the γ parameter in the BN layer as the scaling factor for network pruning. It offers the advantage of avoiding any additional overhead in the network and is an efficient way to implement channel pruning.

## 4. Results

In our experimental setup, we employed the MSTAR standard 10-class classification dataset to showcase the performance of FCCD-SAR. Additionally, we conducted a comparative analysis between FCCD-SAR and existing recognition methods, which reveals the superior performance of the former.

### 4.1. Dataset Introduction and Experimental Settings

#### 4.1.1. MSTAR Dataset

**Figure 8:**
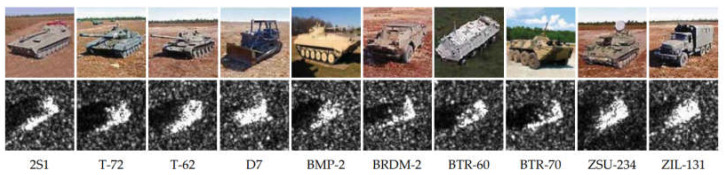
Introduction to the MSTAR dataset.

To precisely evaluate the effectiveness and performance of the proposed model, we utilized the renowned MSTAR dataset, as illustrated in Figure 8. This dataset comprises measured SAR ground stationary target data made available by the MSTAR program and supported by the Defense Advanced Research Projects Agency. It encompasses SAR target images obtained from various vehicle targets at varying azimuths. The MSTAR dataset consists of ten ground targets under standard operating conditions (SOC), including artillery (2S1, ZSU234), armored vehicles (BRDM2, BTR60, BTR70, BMP2, D7, ZIL131), and tanks (T62, T72). Notably, the T72 in the BRDM2 armored vehicle category encompasses three variant types (9563, 9566, C21), while the T72 in the tank category includes three variant types as well (132, 812, S7). Please refer to Table 2 for specific parameters.

#### 4.1.2. SSDD Dataset

The SAR ship dataset SSDD was then selected and used to validate the generalization in comparison experiments to avoid model overfitting. SSDD is the first publicly available dataset specialized in SAR image ship target recognition at home and abroad, which can be used for training and testing to examine the algorithm, and has been used by thirty levels of colleges and research institutes.

**Figure 9:**
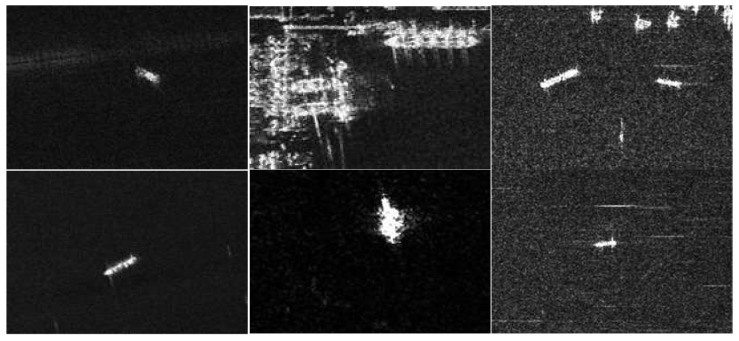
Examples of some of the samples in the SSDD sample.

SSDD was obtained by downloading publicly available SAR images on the Internet and cropping the target area to a size of 640 × 640 pixels and by manually labeling the ship target positions. The dataset is shown in Figure 9. The models were trained using identical parameters, including YOLOV5, a batch size of 16, and a training image size of 640 × 640. The experiments were performed.

### 4.2. Experimental Index

In this experiment, we employ the visual object classes (VOCs) evaluation criteria to assess the recognition performance of our proposed method. To quantify the accuracy of our method, we utilized the average precision at 50% intersection-over-union (mAP50) index. The mAP was calculated by considering both precision (*P*) and recall (*R*) values. Precision measures the proportion of accurate predictions among the samples predicted as positive instances. It was determined based on the number of true positives (*TPs*) and false positives (*FPs*) using the following formula:

(8) $$P=\frac{TP}{TP+FP}$$

The recall is a measure of the probability that the predicted positive instances cover all true positive (*TP*) samples among the actual positive samples. This probability is computed using the *TP* and false negatives (*FN*) according to the following formula:

(9) $$R=\frac{TP}{TP+FN}$$

Accordingly, the *mAP* is determined by computing the integrated area under the precision-recall (*PR*) curve using the given formula:

(10) $$mAP=\int_{0}^{1}P\left(R\right)dR$$

The *F1* score is a commonly employed evaluation metric that provides a comprehensive assessment of the model’s performance by incorporating both precision and recall indicators. It combines accuracy and recall measures to gauge the overall quality of the model:

(11) $$F1=2\times \frac{P\times R}{P+R}$$

In the realm of deep learning, it is customary to evaluate the model’s parameter size, FLOPs, and model volume while designing a model, considering the specific requirements of the application.

(1) Parameters: Parameters serve as a measure of the model’s size, akin to the space complexity of an algorithm. The number of parameters is determined by the video memory size. This encompasses all layers of the model, including convolutional, BN, and fully connected layers, along with the total number of weight parameters in the visual network components.

(2) FLOPs: An abbreviation for floating-point operations, FLOPs represent the quantity of floating-point operations, which can be regarded as a measure of the computational workload. It can be employed to gauge the complexity of an algorithm or model.

### 4.3. Experimental Results

We conducted experimental validation on the MSTAR dataset and performed compatibility experiments to verify the effectiveness and performance of the methods presented in this paper, showcasing the efficacy of each proposed module and improved method. Lastly, we compared our proposed FCCD-SAR with existing SAR target-recognition methods to establish the superior performance of our method.

#### 4.3.1. Comparison of Parameters for CARAFE

We compared different parameters of CARAFE and selected the optimal CARAFE parameters for our method. The results of this comparison are presented in Table 3:

Upon analyzing the data in the table, we observed variations in the number of parameters, FLOPs, and mAP values corresponding to different choices of $k_{encoder}$ and $k_{up}$. After comparing the data in the table, we ultimately selected $k_{encoder}$ as 3, $k_{up}$ as 5 to achieve the optimal results.

#### 4.3.2. Ablation Experiments

Ablation experiments were conducted on the MSTAR dataset to validate the effectiveness of our proposed module and improved method. The experiments focused on the following aspects:

1. Replacing the DarkNet53 backbone of the YOLOV5 benchmark with the more efficient and faster neural network, FasterNet;
2. Substituting the original upsampling operator with the lightweight CA-RAFE upsampling operator;
3. Exchanging the original C3 module with the faster and lightweight C3-Faster module;
4. Incorporating DyHead, a recognition head based on the attention mechanism. The experimental results are presented in Table 4.

From the results of the ablation experiments, the following findings are evident:

1. By adopting the improved and more efficient neural network FasterNet, the FLOP is reduced from 16.0 G to 11.3 G, resulting in a 0.7% improvement in mAP and a reduction in parameters from 7.05 M to 5.57 M. These results validate that our FasterBackbone exhibits superior feature extraction capabilities with fewer parameters and FLOPs. It also proves the correctness of choosing FasterNet as the main network, and FasterNet has a strong lightweight property.
2. Substituting the original upsampling operator with the lightweight CARAFE upsampling operator leads to a 0.6% improvement in mAP, accompanied by a slight increase in FLOPs and parameters. The experimental results demonstrate that CARAFE enhances accuracy while reducing the model’s size. The upsampling operator CARAFE has excellent recognition performance and has the advantage of model lightweight.
3. By replacing the original C3 module with the faster and lightweight C3-Faster module, FLOPs are reduced while maintaining mAP stability. The number of parameters is reduced from 5.86 M to 5.84 M, and the computation is reduced from 12.0 G to 11.9 G. It is also proved that C3-Faster is better than C3 in structure and more in line with the requirements of lightweight.
4. The incorporation of DyHead, a detection head based on the attention mechanism, yields a 0.4% improvement in mAP. After using DyHead, the number of parameters and FLOPs have a small increase, but compared with this, it is an increase of mAP, which meets the expected goal. Experimental results show that the addition of the DyHead attention mechanism can identify more detailed image features.

**Figure 10:**
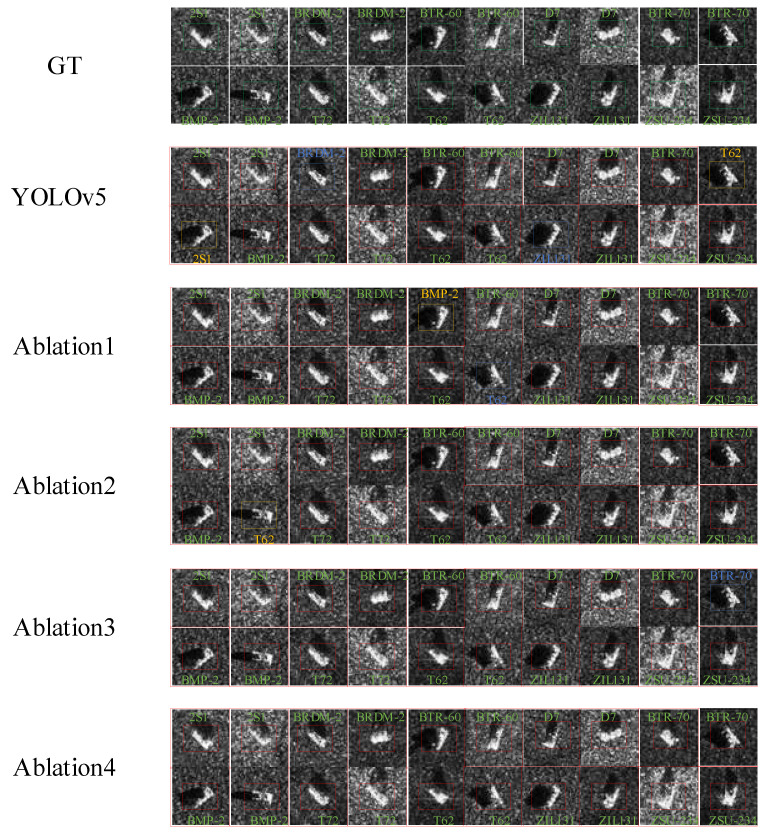
Comparison results of ablation experiment visualization on the MSTAR dataset. The blue rectangular box indicates the missed recognition target, and the yellow rectangular box indicates the false recognition target.

In conclusion, the employed FCCD-SAR method showcases notable improvements in reducing the model size and minimizing FLOPs and parameters, all while maintaining a high level of accuracy. These findings substantiate the lightweight nature of our proposed model. Figure 10 presents the visualization comparison results of the ablation experiment.

#### 4.3.3. Comparison of Network Model Pruning Results

Pruning experiments were performed on the proposed network structure on the MSTAR dataset to compare the pruning results and select the best one.

The experimental results presented in Table 5 demonstrate that as the pruning degree increases, there is a corresponding decrease in the number of parameters and computational requirements. However, accuracy degradation becomes evident when the pruning magnitude exceeds 50%. Therefore, we selected a pruning rate of 50% to strike a balance between achieving a favorable reduction in parameters and computation while maintaining training accuracy.

#### 4.3.4. Comparative Experiment

**Figure 11:**
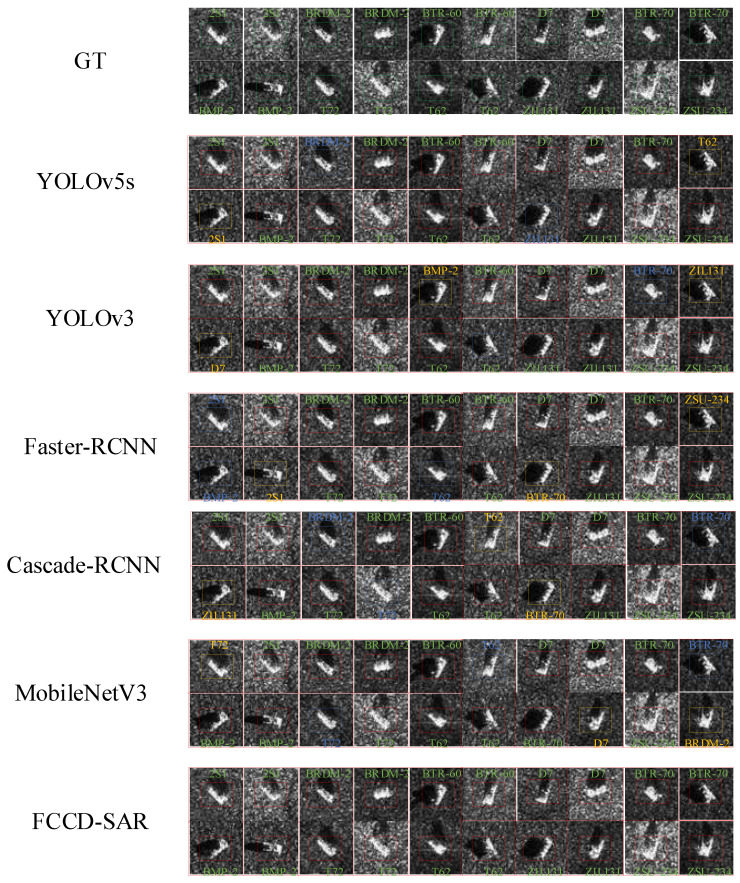
Comparison of visualization recognition results under MSTAR dataset with the latest method. The blue rectangular box indicates the missed recognition target and the yellow rectangular box indicates the false recognition target.

Comparison results with the latest target-recognition methods on the MSTAR dataset: To further validate the robust detectability of our proposed FCCD-SAR model, we conducted experiments to compare it with commonly used single-stage and two-stage target recognition methods on optical images, including YOLOV3, YOLOV5, Faster R-CNN, Cascade-RCNN, and other methods. Compared to the single-stage target recognition method with fewer parameters and lower computational effort, the present experimental method has fewer parameters and less computational effort and is more accurate. Compared to the more accurate two-stage target recognition method, this experimental method is more accurate than it. Meanwhile, this experimental method had fewer parameters and fewer computations, which could satisfy the lightweight requirement. The parameters for each method were set to be approximately the same, ensuring fairness in the comparison experiments. The results are presented in Table 6. Our proposed method achieves the lowest computational and parametric requirements while surpassing the accuracy and exhibiting the highest overall performance among the compared methods. Visualization of the predictions for each recognition method is shown in Figure 11. Our method demonstrated superior target-recognition performance and a lower false recognition miss rate compared to other target recognition methods.

**Figure 12:**
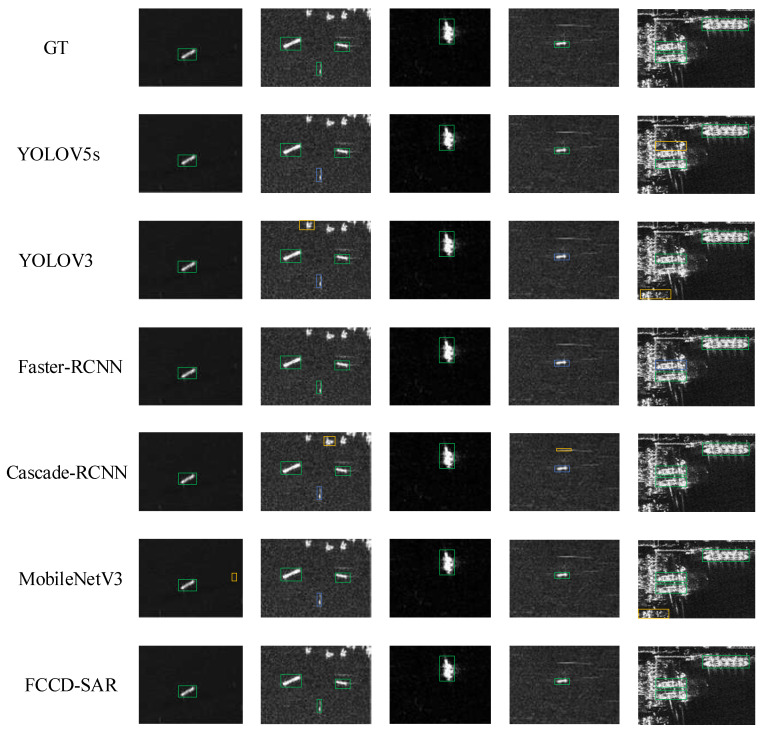
Comparison of visualization recognition results under the SSDD dataset with the latest method. The blue rectangular box indicates the missed recognition target and the yellow rectangular box indicates the false recognition target.

In order to verify the generalization and avoid overfitting, the article added the SAR ship dataset SSDD to corroborate the model performance. Under the SSDD dataset, the experimental results are shown in Figure 12. The experimental results are shown in Table 7. According to the experimental results, it can be found that the model proposed in this paper has certain generalization, and no overfitting occurs, which fully proves the excellent effect of the proposed model.

## 5. Discussion

This paper presented FCCD-SAR, a lightweight algorithm for SAR target recognition based on FasterNet. The method was specifically designed for deployment on embedded devices, taking into consideration the lightweight requirements. Moreover, it incorporates the unique feature information characteristics of SAR images.

In this study, the lightweight benchmark model YOLOv5 was initially introduced, and subsequently, FasterNet, a more efficient and faster neural network, was used to replace the main network. The choice of FasterNet was motivated by its compatibility with the unique characteristics of the SAR image dataset, striking a balance between speed and accuracy.

To minimize both the model size and computational effort, we employed the lightweight upsampling operator CARAFE. CARAFE performs a dot product between the upsampling kernel and the pixels in the surrounding neighborhood of each position within the input feature map. This operation allows for a broader perceptual field during recombination and guides the recombination process using input features. As a result, it enhances the recognition performance of SAR targets while simultaneously reducing the number of parameters.

To improve both the recognition accuracy and computational efficiency of the model, we incorporated the C3-Faster module, which is faster and lighter. This module effectively reduced the number of parameters and computational requirements by selectively discarding unimportant information while maintaining the required level of recognition accuracy.

The attention mechanism-based detection head, DyHead, was incorporated to handle the multi-scale features of SAR targets. DyHead is a dynamic head framework that utilizes an attention mechanism to unify various target detection heads. It leverages attention mechanisms across feature levels for scale perception, spatial locations for spatial perception, and output channels for task perception. By employing this approach, the model’s target detection head achieved enhanced expressiveness and improved target recognition accuracy without increasing the computational effort.

To obtain the optimal model, we employed a pruning technique to reduce the network’s complexity while preserving its accuracy. Subsequently, we evaluated the proposed method on the MSTAR dataset, and the results demonstrated its exceptional performance, achieving an MAP of 99.5%. Notably, the number of parameters was merely 2.72 M, and the FLOPs amounted to 6.11 G, showcasing the model’s efficiency.

## Figures and Tables

**Table 1:** Parameters of FasterBackbone network structure.

	Out Channel	Kernel Size	Stride	Expand
MLPBlock	40	3	1	3
MLPBlock	80	3	1	3
MLPBlock	160	3	1	3
MLPBlock	320	3	1	3
SPPF	1024	3	1	-

**Table 2:** Basic information and experimental settings of experimental datasets.

	Images Size for Training	Train:Valid	Number of Class	Batch Size	Epoch
MSTAR	640×640	8:2	10	16	300
SSDD	640×640	8:2	1	16	300

**Table 3:** Recognition results with various encoder kernel sizes $k_{encoder}$ and reassembly kernel sizes $k_{up}$.

$k_{encoder}$	$k_{up}$	Params (M)	FLOPs (G)	mAP (%)
1	3	7.07	16.1	98.8
1	5	7.08	16.1	98.8
3	3	7.11	16.1	98.9
3	5	7.19	16.3	99.1
5	5	7.21	16.4	99.0

**Table 4:** Results of the ablation experiments on the MSTAR dataset.

Model	FasterNet	CARAFE	C3-Faster	DyHead	Params (M)	FLOPs (G)	mAP (%)
YOLOV5s	×	×	×	×	7.05	16.0	98.1
FCCD-SAR	√	×	×	×	5.57	11.3	98.8
FCCD-SAR	√	√	×	×	5.86	12.0	99.1
FCCD-SAR	√	√	√	×	5.84	11.9	99.1
FCCD-SAR	√	√	√	√	6.00	12.2	99.5

**Table 5:** Pruning results for FCCD-SAR.

Method	Params (M)	FLOPs (G)	mAP (%)
FCCD-SAR (Baseline)	6.00	12.2	99.5
FCCD-SAR (20%Pruned)	5.12	10.98	99.5
FCCD-SAR (40%Pruned)	3.91	8.54	99.5
FCCD-SAR (50%Pruned)	3.34	7.32	99.5
FCCD-SAR (60%Pruned)	2.72	6.11	99.2
FCCD-SAR (80%Pruned)	1.50	3.66	98.6

**Table 6:** Comparison with the latest target-recognition methods on the MSTAR dataset.

Method	Params (M)	FLOPs (G)	P (%)	mAP (%)	Inference Time (ms)
YOLOV5s	7.05	16.0	97.8	98.1	4.1
YOLOV3	8.7	13.0	96.9	97.2	5.7
Faster-RCNN	41.12	91.41	96.5	96.4	82.6
Cascade-RCNN	69.17	119.05	96.9	96.8	98.5
MobileNetV3	4.29	7.2	96.3	96.6	4.4
FCCD-SAR	2.72	6.11	99.5	99.5	3.4

**Table 7:** Comparison with the latest target-recognition methods on the SSDD dataset.

Method	Params (M)	FLOPs (G)	P (%)	mAP (%)	Inference Time (ms)
YOLOV5s	7.06	16.2	96.8	97.8	4.2
YOLOV3	8.9	13.3	96.2	96.8	6.7
Faster-RCNN	44.34	92.11	95.2	94.3	85.1
Cascade-RCNN	71.32	120.48	96.1	95.9	100.8
MobileNetV3	4.58	8.2	93.5	93.6	5.3
FCCD-SAR	3.32	7.4	98.8	98.7	3.6

## Data Availability

The original data will be shared at the author’s discretion. Please contact the corresponding author directly.
